# Breaking the Excitation-Inhibition Balance Makes the Cortical Network's Space-Time Dynamics Distinguish Simple Visual Scenes

**DOI:** 10.3389/fnsys.2017.00014

**Published:** 2017-03-20

**Authors:** Per E. Roland, Lars H. Bonde, Lars E. Forsberg, Michael A. Harvey

**Affiliations:** ^1^Faculty of Health Sciences, Center for Neuroscience, University of CopenhagenCopenhagen, Denmark; ^2^Department of Physiology, University of FribourgFribourg, Switzerland

**Keywords:** cerebral cortex, mechanics of vision, dynamical systems, balanced network, inhibition, voltage sensitive dye

## Abstract

Brain dynamics are often taken to be temporal dynamics of spiking and membrane potentials in a balanced network. Almost all evidence for a balanced network comes from recordings of cell bodies of few single neurons, neglecting more than 99% of the cortical network. We examined the space-time dynamics of excitation and inhibition simultaneously in dendrites and axons over four visual areas of ferrets exposed to visual scenes with stationary and moving objects. The visual stimuli broke the tight balance between excitation and inhibition such that the network exhibited longer episodes of net excitation subsequently balanced by net inhibition, in contrast to a balanced network. Locally in all four areas the amount of net inhibition matched the amount of net excitation with a delay of 125 ms. The space-time dynamics of excitation-inhibition evolved to reduce the complexity of neuron interactions over the whole network to a flow on a low-(3)-dimensional manifold within 80 ms. In contrast to the pure temporal dynamics, the low dimensional flow evolved to distinguish the simple visual scenes.

## Introduction

How do neurons in the brain collaborate to produce visual perception? This question has no satisfactory answer yet. The idea of neuron coding in vision builds on the notion that the temporal sequence of spikes from single neurons or a population of neurons is sufficient to distinguish different visual objects or scenes (Barlow, 1961; Gross et al., 1972; Richmond and Optican, 1990). The spike trains are analyzed statistically or analyzed for their information content to find a match or correlation with the present scene or object. An alternative to these objectives is to use a dynamical systems approach and examine the evolution of the spiking in (abstract) state space. Forsberg et al. (2016) used both approaches, i.e., examined the temporal dynamics of spiking populations of neurons in the primary visual cortex in state space and examined the spike rates in single trials from the same population. Although both the temporal spike rate changes and the temporal spiking dynamics in state space reported whether a sharp or a smooth visual transient changed the visual scene, neither could differentiate simple visual scenes on a single trial basis. When one examines temporal structures of spike trains or membrane currents for information about a current visual scene and when one analyzes the temporal aspects of these variables in state space, one neglects what goes on in the remaining network, and one neglects the possibility that combinations of temporal and spatial evolution of spiking or membrane potential changes might be more efficient in distinguishing visual scenes.

Therefore, we examined the space-time dynamics of the membrane potential changes of the visual areas 17, 18, 19, and 21 of the ferrets used by Forsberg et al. (2016). By space-time dynamics, we mean the progression of membrane current changes in the topological space made up of the cortical network of neurons. The cortical network of neurons is formed by the synaptic connected neurons having their cell bodies in the cerebral cortex. The evolutions of the membrane potentials over the four visual areas are described as trajectories and vectors in (mathematical abstract) state space. We found that within 50–90 ms after exposure to the visual scenes, the space-time dynamics reduced to a 3-dimensional state space where the trajectories of the membrane potential changes caused by the individual scenes clearly separated. These diverging flows on a low dimensional manifold show that the connectivity and spiking dynamics in the visual cortical network swiftly constrain the patterns of membrane potential changes to simple low dimensional solutions unique for each of the scenes. Although this succinctly describes the space-time interactions of the membrane potential changes in mathematical terms, it does not provide a biophysical explanation of the mechanisms.

The visual scenes were very simple, composed of one stationary or moving bar. Creutzfeldt et al. (1974) showed that a moving bar first depolarized and then hyperpolarized the neurons in the primary visual cortex of the cat. Currently there seems to be a consensus that neurons in the primary visual cortex of carnivores and primates have a balance between excitation and inhibition during visual stimulation (Destexhe and Pare, 1999; Anderson et al., 2000; Monier et al., 2003, 2008; Priebe and Ferster, 2005; Haider et al., 2006; Rudolph et al., 2007; Tan et al., 2011; Xue et al., 2014; Dehghani et al., 2016). The terms excitation and inhibition are used in several ways. In this context, balance between excitation and inhibition in the cortical network means that the excitatory membrane currents = inhibitory membrane currents (van Vreeswijk and Sompolinsky, 1996). Alternatively the balance has been interpreted as balanced ratio between excitatory and inhibitory membrane conductances (Denéve and Machens, 2016 for a recent review). It does not matter too much which of these definitions of balance one adheres to, the point is that the balance is not instantaneous. If it were, there would be no excitatory spiking of the neurons. The membrane potential, however, cannot show the balance between excitatory and inhibitory membrane currents, because the membrane potential can be high during large inhibitory currents and conductances and currents can change markedly without affecting the membrane potential too much (Anderson et al., 2000; Monier et al., 2003, 2008; Priebe and Ferster, 2005; Haider et al., 2006; Rudolph et al., 2007; Okun and Lampl, 2008; Tan et al., 2011; Xue et al., 2014). The finding of Creutzfeldt et al. (1974) is still valid, although it does not say anything about the instantaneous balance between excitation and inhibition. The time derivative of the membrane potential reports this balance, because it equals the sum of the ionic currents through the membrane divided by the specific capacitance (the specific capacitance is regarded constant) (Equation 2, Methods). If the inhibition first increases after a delay, there would be a chance that excitation can spread through the cortical network. The question is how big this delay is.

*In vivo* patch clamp recordings of individual neurons in primary visual cortex have generally shown two types of responses. The excitatory conductance increased while the inhibitory conductance decreased or did not change initially, but thereafter increased with a delay of some 100–400 ms. For other stimuli and for other neurons, the excitatory and inhibitory conductances increased almost simultaneously; or the inhibitory conductances lagged the excitatory conductances with a small 10–20 ms delay (Anderson et al., 2000; Monier et al., 2003, 2008; Priebe and Ferster, 2005; Haider et al., 2006; Rudolph et al., 2007; Okun and Lampl, 2008; Tan et al., 2011; Xue et al., 2014). In these studies, the excitatory (inward) currents and inhibitory (outward) currents were measured in the cell bodies of only a limited number of neurons (typically 25–40). As cell body membranes only make up an estimated 2% of the total membrane surface of cortical neurons (Fiala and Harris, 1999), such measurements say little about the balance of excitatory and inhibitory currents in the dendrites and axon terminals of the measured neurons (Williams and Mitchell, 2008).

In this report, we take a network approach and examine the space-time dynamics of the membrane potential changes with a voltage sensitive dye (VSD) (Cohen et al., 1974) simultaneously in areas 17, 18, 19, 21. The cortical surface exposed for measurement was 12 mm^2^ (assuming 70,000 neurons/mm^3^ and that 50% of these contribute fluorescence to the VSD signal, VSD(t), the estimate of the number of neurons measured would be around 400,000). Voltage sensitive dyes reliably report changes in membrane potential of neurons (Cohen et al., 1974). The voltage sensitive dye signal, VSD(t), reports relative increases and decreases of population membrane potential compared to baseline at the network scale and without any bias toward spiking probabilities, receptive fields and stimulus feature preferences (Berger et al., 2007). Under the assumption that the membranes in a small volume of the cortical network from where the fluorescence originates can be regarded as one compartment, the dVSD(t)/dt of that compartment is the sum of the membrane currents multiplied by a constant (Eriksson et al., 2008) (Equation 3, Methods). The dVSD(t)/dt thus reports the instantaneous balance between excitatory currents and inhibitory currents in the network (Methods). If the balance changes toward net inhibition (no matter whether this is due to reduced excitatory currents or increased inhibitory currents) the dVSD(t)/dt significantly decreases below baseline fluctuations. Thus, we label significant decreases of the dVSD(t)/dt as net inhibition, NINH, and significant increases as net excitations, NEX.

The dVSD(t)/dt has been used to measure excitation-inhibition balance in the cortex (Eriksson et al., 2008; Harvey et al., 2009; Onat et al., 2011a; Harvey and Roland, 2013). Onat et al. (2011a) showed that the balance between excitation and inhibition in response to a drifting grating, at 100 ms goes into NINH and then switches to NEX. This and the other dVSD(t)/dt studies showed the temporal characteristics of the excitation- inhibition balance at one location or averaged from all locations. However, to our knowledge, the space-time evolution of inhibition and the balance between excitation and inhibition has not been explored.

NEX and NINH evolved with similar space-time dynamics in all four visual areas and distinguished the visual scenes. The visual stimuli disrupted the tight balance between excitation and inhibition and drove the network into a regime tolerating large amplitude NEX and NINHs. However, at each cortical spot in the four visual areas, the amount of integrated NEX and NINH were strongly correlated, showing that net-excitation after longer delays became balanced with net-inhibition overall in the exposed part of the cortical network.

## Materials and methods

### Animals

The voltage sensitive signals VSDi(t) were measured in 8 female adult ferrets. These animals also participated in the study of Harvey and Roland (2013) but none of the results presented in this study have been presented in Harvey and Roland (2013) or elsewhere. Laminar action potentials were measured at the border between areas 17/18 in 10 adult female ferrets of which 6 also had their VSD_i_(t) measured. The Regional Ethics Committee of Stockholm had approved all procedures. The ferrets were anesthetized and artificially ventilated with 1% isofluran mixed with N_2_O:O_2_, 1:1 and paralyzed with pancuronium bromide throughout the experiments. A left-sided craniotomy with a chamber affixed to the skull exposed cortical areas 17, 18, 19 and 21. The end-expiratory partial pressure of CO_2_, pCO2, was maintained between 3.5 and 4.5 KPa.

### Stimuli

In all animals, known cortical landmarks were used to guide a single electrode penetration at the estimated crossing of the vertical and horizontal meridian in areas 17/18. The receptive field (RF) at this area was then mapped using an m-sequence method (Reid et al., 1997). The monitor position was then adjusted to be precisely centered to this RF location.

Stimuli were presented in a pseudorandom order on a video monitor with a refresh rate of 144 Hz located 57 cm in front of the animal (Cambridge Research Systems, Kent UK). Stimuli consisted of 1° × 2° horizontal bars (64.5 cd m^−2^) on a homogenous gray background (7.2 cd m^−2^). There were six conditions: (1) A bar moving downwards from the center of field of view (CFOV) with a velocity of 25.4° s^−1^ for 413 ms along the vertical meridian. (2) A bar moving upward from the CFOV with a velocity of 25.4° s^−1^ for 413 ms along the vertical meridian. (3) Upward and (4) downward moving bars initially flashed 10.5° below and 10.5° above the center of field of view (CFOV) respectively and moving a total of 21° with a velocity of 25.4° s^−1^ along the vertical meridian for a period of 825 ms with start and end points equidistant from the screens center. (5) A single stationary bar was presented at the CFOV for 250 ms. In addition (6) a no-stimulus condition was included, consisting of a homogenous gray screen (7.2 cd m^−2^) presented continuously in between the stimulus conditions. Each of these six conditions was repeated 50 times.

### Voltage sensitive dye measurements

The procedures were described in detail recently (Harvey and Roland, 2013).

The cortex was stained for 2 h with the voltage sensitive dye RH1838 (0.53 mg ml^−1^; *n* = 3) or RH1691 (0.53 mg ml^−1^; *n* = 5) (Optical Imaging, Rehovot, Israel). Imaging was centered on the initial recording site and acquired using a 464-channel photodiode array, (H469-IV WuTech Instruments Gaithersburg, MD) Images of the voltage sensitive dye signal were acquired at a rate of 1.6 kHz, stimulus presentation was synchronized to the electrocardiogram, and respiration stopped during stimulus presentation. The voltage sensitive dye signal, VSD(t), in most animals (*n* = 7) covered both the representations of the CFOV at the 17/18 border and the CFOV representation at 19/21 border.

### Laminar electrophysiology

#### A: ferrets having the VSD(t) measured

The multiunit activity was recorded with single shank, 16 channel, laminar probes (NeuroNexus, Ann Arbor, MI) with recording site resistances of 2–3 MΩ, and leads separated by 100 μm. The signal was digitally band pass filtered between100 Hz and 10 KHz. At each recording site, receptive fields were first mapped using the methodology noted above. For electrode penetrations in cortex mapping the CFOV, the receptive fields in the granular layer were less than 3°.

#### Current source density

We estimated current source densities from the second spatial derivative of the local field potentials in filtered (0.5–600 Hz) original data files (Rappelsberger et al., 1981). The data files were averaged over 50 trials for each lead either from a single stationary bar or from whole screen flashes of 1 Hz. Layer 4 was estimated to start where the early sink appeared (the sink being at the upper part of layer 4), the supragranular and infragranular layers above respective below layer 4 (Harvey et al., 2009).

### Data analysis voltage sensitive dye

The voltage sensitive dye signals were analyzed up to 800 ms after the start of the stimulus. The details of the analysis have been described elsewhere (Roland et al., 2006; Eriksson et al., 2008; Harvey and Roland, 2013). The signal in the blank (background alone) condition was subtracted from the signal of the stimulus conditions and divided by the background fluorescence to yield the fractional fluorescence (ΔF/F_0_) referred to here as VSD_i_(t). Usually the VSD_i_(t) of 8–12 trials were averaged to give one time-series VSD(t).

#### Cytoarchitectonics

**Figure 1:**
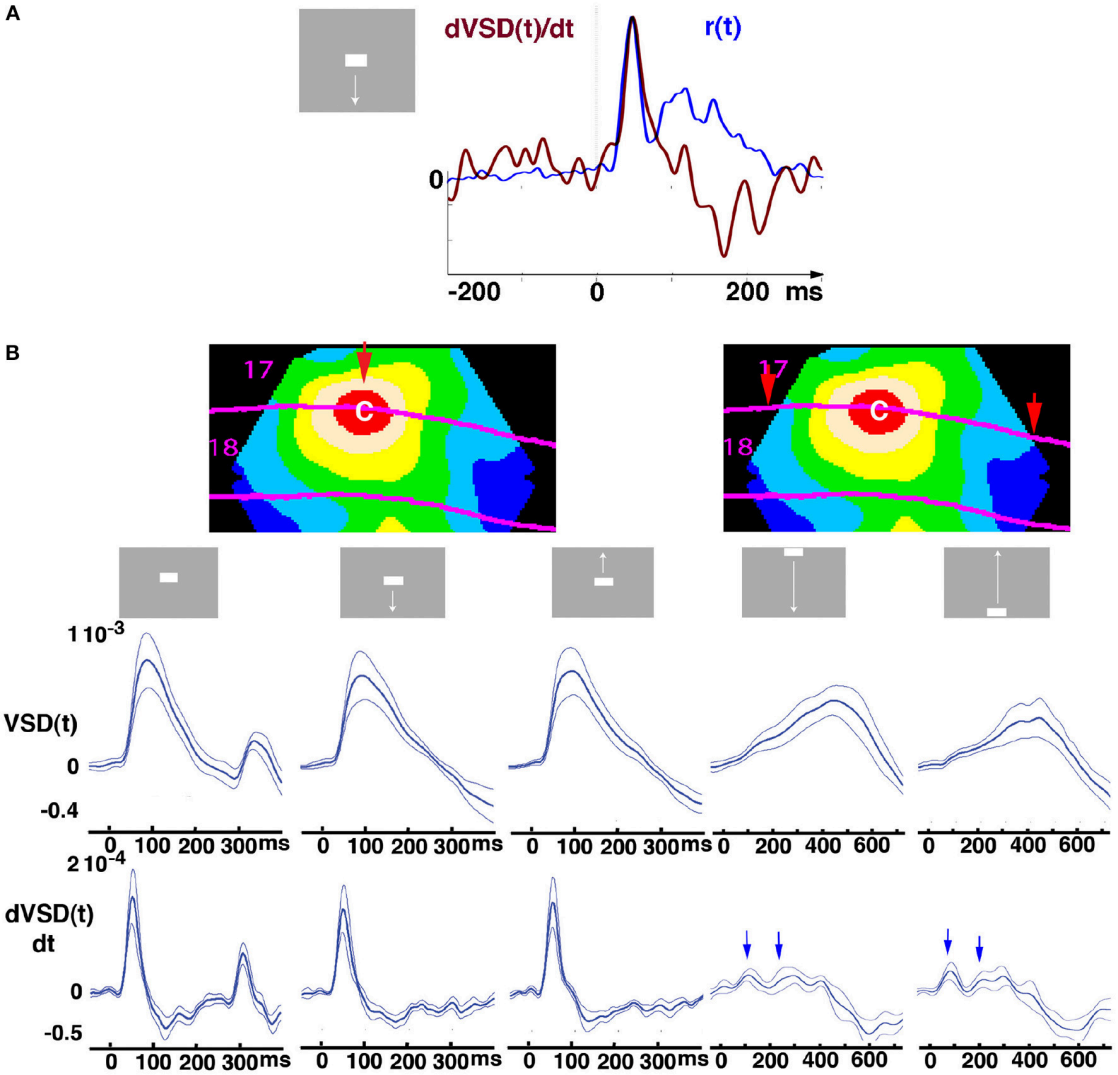
**Original averaged data from the cortex at the red retinotopic zone for the CFOV. (A)** Relation between dVSD(t)/dt (peak value 4.2 10^−5^) in upper layers (red curve) and supragranular MUA spiking rate (peak value 41 Hz) from cortex mapping the CFOV; (Animal 4, penetration 6). **(B)** The contour plots are identical snapshots of the mean VSD(t) of 6 animals at absolute maximum (103 ms, stationary bar stimulus) to show the red sampling area mapping the CFOV from where the temporal course of the mean VSD (t) and mean dVSD(t)/dt were sampled. The red arrow (left) marks the location where the mapping of the bar started for the 3 stimuli initially presented at CFOV. The 2 arrows (right) mark where the mappings of the bars moving from the peripheral FOV started. Stimulus start 0 ms. The stationary square displayed for 250 ms had an OFF response, moving bars did not. Mean ± standard error of mean (SEM) shown (*n* = 7 animals) for respective stimulus (top). Bars starting in the C-zone evoked sharp net excitatory transients followed by net inhibitions, shown in the dVSD(t)/dt plots; whereas the bars moving toward the CFOV evoked early and late net excitations (blue arrows), which after the bar had been mapped at cortex representing the CFOV (after 450 ms) was followed by net inhibition.

Six of the eight ferrets had their cytoarchitectural borders determined between areas 17 and 18, 18 and 19 and 19 and 21. Their brains were sectioned, stained and the cytoarchitectural borders determined. The sections were reconstructed to a volume and the borders overlaid the hexagonal map of the VSD(t) (**Figure 4**) as described in detail in Harvey and Roland (2013). The individual VSD(t) with the cytoarchitectural borders were aligned as described in detail in Harvey et al. (2009), to produce the average cytoarchitectural borders in Figures 1, **3**, **4** and Movies 1–7.

#### State space analysis of dVSD(t) trajectory differences in the time domain

In each animal, the 4 photodiode channels sampling from the cortex mapping the CFOV, were chosen. The mean of dVSD(t)/dt from these 4 channels in one animal is a time vector for one stimulus condition. A principal component analysis, PCA, was then performed on the data matrix for the 2 stimulus conditions to be compared in state space for all 8 animals. The first 3 components accounted for 88–97% of the total variance. For each condition and animal, the first three principal components were used as regressors against the time vector of the condition to obtain a beta-estimate for each principal component, showing how well it describes the condition. The polynomial of principal components times the beta-estimates was then calculated to show how a particular condition is described by all three principal components. The difference between two conditions' polynomials within an animal was further calculated to describe how the two conditions differ in the same 3-dimensional state space as a function of time. Features in the time series of the conditions that are not part of the common 3-dimensional state space are thus effectively removed and thereby not compared between conditions. Across animals, a statistical *t*-test was used to test if the null-hypothesis “no difference between conditions” in any given time point could be rejected in the given state space. False Discovery Rate was finally used to correct for multiple comparisons over time (FDR at 0.05).

#### Net excitation and net inhibition

Experimentally the change in the VSD(t) is proportional to the change in the average membrane potential Vm(t) of a small population of neurons (Petersen et al., 2003; Ferezou et al., 2006). Thus:

(1) $$\text{dVSD}(\text{t})/\text{dt }\approx \text{k }{\text{dV}}_{\text{m}}(\text{t})/\text{dt}$$

The change in the membrane potential is the sum of the ionic currents divided by the specific capacitance:

(2) $$\begin{array}{l} {\text{dV}}_{\text{m}}(\text{t})/\text{dt}=-1/{\text{C}}_{\text{m}}\;[{{\text{g}}_{\text{Na}}}^{+}({\text{V}}_{\text{m}}(\text{t})-{\text{E}}_{{\text{Na}}^{+}})+{{\text{g}}_{\text{Ca}}}^{2+}({\text{V}}_{\text{m}}(\text{t})-{{\text{E}}_{\text{Ca}}}^{2+})\\ \;+\;{{\text{g}}_{\text{Cl}}}^{-}({\text{V}}_{\text{m}}(\text{t})-{{\text{E}}_{\text{Cl}}}^{-})+{{\text{g}}_{\text{K}}}^{+}({\text{V}}_{\text{m}}(\text{t})-{{\text{E}}_{\text{k}}}^{+})] \end{array}$$

In which C_m_ is the specific capacitance of these membranes, E is the reversal potential, and g the conductance. Then under the assumption that the membranes in a small volume of the cortex from where the fluorescence originates can be regarded as one compartment (Eriksson et al., 2008; Roland, 2010), the dVSD(t)/dt of that compartment is:

(3) $$\begin{array}{l} \text{dVSD}(\text{t})/\text{dt}\approx \;-\text{ k}/{\text{C}}_{\text{m}}[{{\text{g}}_{\text{Na}}}^{+}({\text{V}}_{\text{m}}(\text{t})-{\text{E}}_{{\text{Na}}^{+}})+\;{{\text{g}}_{\text{Ca}}}^{2+}({\text{V}}_{\text{m}}(\text{t})-{{\text{E}}_{\text{Ca}}}^{2+})\\ \;+\;{{\text{g}}_{\text{Cl}}}^{-}({\text{V}}_{\text{m}}(\text{t})-{{\text{E}}_{\text{Cl}}}^{-})+{{\text{g}}_{\text{K}}}^{+}({\text{V}}_{\text{m}}(\text{t})-{{\text{E}}_{\text{k}}}^{+})] \end{array}$$

At the network scale, at which our measurements of the dVSD(t)/dt were done, the membrane compartment comprises the membranes of thousands of neurons at a single spot. Although a few of these neurons may be depolarized over their spiking threshold, these action potentials will not be detectable in the VSD(t) (Grinvald et al., 1994; Arieli et al., 1995; Senseman, 1996; Roland, 2002; Slovin et al., 2002; Petersen et al., 2003; Jancke et al., 2004; Ferezou et al., 2006; Roland et al., 2006; Ahmed et al., 2008; Eriksson et al., 2008; Harvey et al., 2009; Ayzenshtat et al., 2010; Chavane et al., 2011; Harvey and Roland, 2013; Müller et al., 2014). The reason most likely is that the fraction of membrane depolarized over spiking threshold is too minute. Consequently, the VSD(t) will reflect a population membrane potential above the reversal potentials for Cl^−^ and K^+^ currents and below the reversal potentials for Na^+^ and Ca^2+^. The dVSD(t)/dt, thus indicates the (weighted sum of) membrane currents of the membranes in the compartment.

Definition of net excitation and net inhibition: For a single spot in the network where the dVSD(t)/dt is measured a significant decrease from baseline fluctuations of dVSD(t)/dt is a net inhibition, NINH(t) and a significant increase is a net excitation, NEX(t).

The baseline fluctuations were determined in the pre-stimulus period. Statistically significant decreases and increases from baseline fluctuations *p* < 0.05 (see below).

For the analysis of net-excitation and net-inhibition, the VSD(t) was filtered with a temporal Gaussian filter σ = 40 ms and then the temporal derivative dVSD(t)/dt was calculated (Matlab®). A filter of σ = 12 ms did not change the results. No spatial filtering was done, but in the figures and movies the original hexagon pixels were sub-sampled.

### Statistical significance

In each animal, statistical comparisons between the pre-stimulus time interval and the post-stimulus interval for all 464 photodiode channels are corrected for mass significance with a false discovery rate of 0.05 (Benjamini and Hochberg, 1995). The results are movies and snapshot sequences of only statistically significant dVSD(t)/dt increases and decreases (*p* < 0.05). As our definition of NEX and NINH is significant increases and decreases from baseline fluctuations in the dVSD(t)/dt in the pre-stimulus period, it implies that these fluctuations will be smaller than the NEX(t) and NINH(t), and that there might be periods in the post-stimulus interval in which NEX(t) and NINH(t) are not defined. Non-statistically significant parts of the cortex will have the same color as the pre-stimulus interval in figures and movies. Movies 4–7 and **Figures 3**, **4** show the spatio-temporal evolution of the NEX(t) and NINH(t) (FDR at 0.05).

### State space analysis of dVSD(t)/dt trajectories

**Figure 2:**
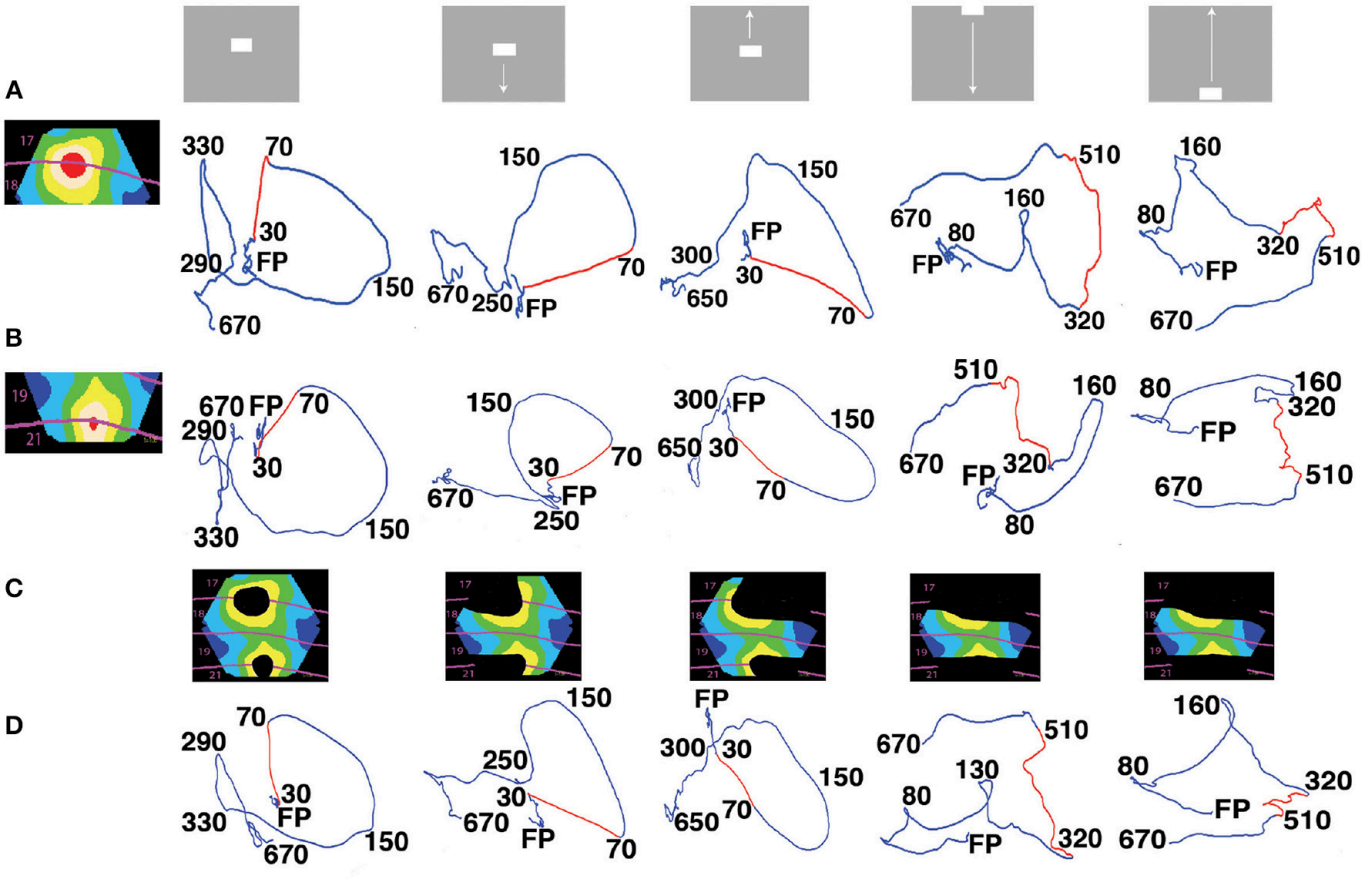
**State space trajectories of the dVSD(t)/dt in different cortical zones are similar. (A)** The 5 visual scenes and their associated trajectories in 3-dimensional state space (Methods) from the red zone. Each trajectory is a projection of the first 3 principal components (for all 8 animals) over time shown in ms after the stimulus start by the black numbers. FP is the origo of the state space (fixed-point). The first row of trajectories comes from the red zone, i.e., the cortex mapping the CFOV. The red parts of the trajectories show the time intervals with the largest acceleration. **(B)** Trajectories from the red (19/21 area) zone also mapping the CFOV. Red parts of trajectories show the fastest acceleration for the bars initially mapped in cortex representing CFOV; the last two red parts are shown for comparison with A. **(C)** Cortical zones in which the stimulus in the top row was *not* mapped according to retinotopy and active evoked spiking. **(D)** The dVSD(t)/dt trajectories for each of the non-mapping zones in **(C)**. Red parts of trajectories only shown for comparison with **(A,B)**. In all zones the trajectories were significantly different (FDR at 0.05) from the pre-stimulus period in the time interval 40–200 ms for conditions with stimulus starting in CFOV and for interval 105–525 ms for the last 2 conditions. Notice the similarities in trajectories irrespective of whether the stimulus was mapped by retinotopic localized spiking or not.

To produce Figure 2, The dVSD(t)/dt time-series underwent PCA. For each of the 6 cortical zones in the figure, the data matrix comprised 6 conditions. The PCA was done using Singular Value Decomposition as implemented in the Matlab® PCA routine.

(4) $$X=U\Sigma V^{\text{T}},\;X\in {\mathbb{R}}^{N\times L}$$

where [*N* = *N*_*times*_ · *N*_*animals*_ · *N*_*conditions*_ = 1, 000 · 8 · 6 = 48, 000] is the number of observations and *L* is the number of photodiode channels (for example 15 if the zone of cortex was monitored by 15 channels). When all of the exposed cortex was examined, *L* = 464 (corresponding to the 464 photodiode channels monitoring all 4 cortical areas).

The vectors **U** = [*u*_1_*u*_2_*u*_3_ … *u*_*N*_] hold the coordinates of the observations in the PCA vector space spanned by ${\text{V}}^{\text{T}}={\left[v_{1}v_{2}v_{3}\ldots v_{L}\right]}^{\text{T}}$. The diagonal matrix $\Sigma =\left[\begin{array}{ccc} \sigma_{1} & 0 & 0\\ \; & \ddots & \;\\ 0 & 0 & \sigma_{L} \end{array}\right]$ holds the singular values which scales vectors in **U** to reflect the variance explained by each of the *L* principal components. The state-space trajectories in 3 dimensions are the (*u*_1*i*_, *u*_2*i*_, *u*_3*i*_) coordinates, plotted over a time series of i's (Figures 2, **5**, Movies 8–14). Note that the (*u*_1*i*_, *u*_2*i*_, *u*_3*i*_) ≠ (*x*_*i*1_*x*_*i*2_*x*_*i*3._). Figure 2 shows the trajectories of the first three components.

### Balance of net excitation net inhibition and delay

To examine the balance between NEX and NINH we calculated the time-averages, E(NEX) and E(NINH) of the NEX(t) and NINH(t) for the post-stimulus time intervals in which NEX(t) and NINH(t) were defined in the post-stimulus period from 0 to 800 ms. We calculated the integrals of NEX(t) over the time interval the NEX(t) was defined, added the integrals and divided by the number of time frames in the interval the NEX(t) was defined. Similar for NINH(t).

This produced the hexagon maps of E(NEX) and E(NINH) in **Figure 6**.

Thereafter we determined the delay between the maximum NEX, NEXmax; and minimum of NINH, NINHmin, in all pixels where NEX(t) and NINH(t) were defined for each condition in each animal and calculated a mean delay for that condition in each animal.

We also calculated the correlation between E(NEX) and E(NINH) pixel by pixel for each condition and animal (**Figure 6**).

### NEX and NINH velocities

The NEX and NINH velocities of propagation were determined by the wave-front algorithm of Ahmed et al. (2008). As the algorithm assumes that the parameter amplitude progresses as an increase (see Figure 3 in Ahmed et al., 2008) the –NINH(t) = −dVSD(t)/dt file was used. Shortly, a region of interest was made covering the progress of the NEX or NINH in an animal. The mean dVSD(t)/dt for the time interval chosen, dVSD (respective −dVSD(t)/dt) was calculated. Then all values of dVSD(t)/dt > dVSD were divided in 10 amplitude levels and the cortical positions (mm) of these amplitude levels vs. time (ms) were plotted and a linear regression was made giving the velocity in mm/ms and its statistical significance determined (in this paper < 0.01; **Figure 7**. For further details see Ahmed et al. (2008). The algorithm, thus, does not require a progress with constant amplitude and velocity.

### NEX and NINH phase lags between areas

We used the algorithm of Müller et al. (2014). This algorithm makes a Hilbert transform of the data under the assumption that all parts of the cortex monitored can be seen as one wave of NEX or one wave of NINH. The different positions on the cortex, x, and hence the different cortical areas will be in different phases if there are delays in the propagation of NEX or NINH between areas. The real part at position x is dVSD_*x*_(t) = Re (x, t). The phase, z = x + iy, is a complex number.

The algorithm calculates the imaginary part as:

(5) $$Im\;\;z\;(x,t)=\;P\underset{-\infty }{\overset{\infty }{\int }}\frac{dt^{\prime }}{\pi }Re\frac{z\;(x,t^{\prime })}{t-t^{\prime }}$$

from this the phase

(6) $$\theta \;\left(x,t\right)=tan^{-1}\;\left(\;\frac{Im\;z\;(x,t)}{Re\;z\;(x.t)}\right)=tan^{-1\;}\left(\frac{Im\;Z\;(x,t)}{dVSD\left(x,t\right)}\right)$$

giving the phase. Phase delays between the earliest point x_0_ and the other x_*s*_ are then calculated in ms (**Figure 8**).

To visualize the phase in Movie 15 of the VSD(t), the VSD(t) was normalized to the maximum VSD(t) value in the post-stimulus time interval, VSDmax, i.e., pixel values were VSD(t)/VSDmax.

### The spatio-temporal evoked spiking in areas 17 and 18, 19, and 21

The positions of the electrode penetrations were transferred to a map of the cortex monitored by the photodiode-array. The electrode penetrations preserved their relation to the cytoarchtectural border between areas 17 and 18. The photo-diode cortical map was centered to the electrodes mapping the very center of the field of view (see *Stimulation* section). The electrode penetrations are marked with white circles in the movies.

The treatments of the spike data and the calculation of the proportion evoked trials ms by ms for each electrode penetration was done separately for supragranular, granular, and infragranular layers as described in Forsberg et al. (2016).

For each electrode penetration the proportion of *evoked trials* (maximum 50 trials) was calculated for the specific layer. Evoked trials of spiking are trials in which the spiking trajectories are outside the separatrix separating the spontaneous states from the evoked spiking states in state space (for state-space analysis of spiking see Forsberg et al., 2016). First episodes of a single spiking trial were detected as evoked if the velocity vectors of the trajectory of the spiking in state space were located outside the separatrix (threshold) separating the state space into spontaneous ongoing spiking and evoked spiking (for details see Forsberg et al., 2016). The proportion of trials evoked is shown color-coded for each ms for each penetration. The number of encircled positions is less than 82, because one position may represent more than 1 electrode penetration (up to 3), and a few electrode positions were outside the map. (Movies 1–3).

## Results

In the experiments, the anesthetized ferrets were exposed to a gray screen in an otherwise dark room. The scene suddenly, within 7 ms, shifted and a continuously moving bar, or occasionally a stationary bar appeared (Figure 1). We measured the voltage sensitive dye signal, VSD_i_(t), simultaneously from all points of the exposed cortex comprising visual areas 17, 18, 19, and 21. The multi-unit activity, MUA(t), was also measured from all layers in areas 17 and 18 to the same stimuli; albeit not simultaneously with the VSD_i_(t) measurements. The VSD_i_ data (Methods) presented in this report were averaged over 8–12 trials belonging to one stimulus condition to give the averaged signal, VSD(t) and the dVSD(t)/dt (Figure 1). Figure 1A shows the typical time relation between the average spike rate and the time derivative d(VSD(t))/dt. For the scenes with stimuli appearing in the center of field of view, CFOV, the dVSD(t)/dt shows a strong NEX transient at the arrival of the increased spiking from layer 4 (Movies 1, 3) and thereafter NINH. For the scenes, in which the stimulus passed through the CFOV after 450 ms, the network at the retinotopic site for the CFOV showed two smaller NEXes prior to the passage (arrows), followed by a smooth further NEX that at 450 ms was replaced by a NINH (Figure 1B). The fluctuations of the dVSD(t)/dt in the pre-stimulus period were significantly smaller than the deviations caused by the changes of the visual scene (FDR at 0.05, Methods).We interpret these smaller fluctuations as reflecting a tight balance between inhibition and excitation.

### Temporal dynamics of dVSD(t) in different cortical zones

When stimuli are presented in the center of field of view, they first increase the spiking in a small zone at the border between areas 17 and 18 and then, with a delay, in a similar zone at the border between areas 19 and 21 (Roland et al., 2006; Harvey et al., 2009). First we examined the space-time dynamics of the balance between excitation and inhibition, dVSD(t)/dt, inside and outside zones in the network where the bars were retinotopically mapped. We used a dynamical systems approach (Huys et al., 2015; Forsberg et al., 2016) and examined the evolution of the dVSD(t)/dt for each zone in multidimensional state space (Methods). In this state space, the time evolution of the dVSD(t)/dt evolves as a trajectory. Each coordinate of the trajectory corresponds to one state of the network in one zone. Figure 2A shows the temporal dynamics of the derivative of the dye signal, dVSD(t)/dt for the two retinotopic sites mapping the CFOV in areas 17/18 and areas 19/21. Prior to stimulus onset, the trajectories evolve in a narrow part of state space close to (0,0,0) (marked with FP in Figure 2). When the stimulus drives the evolution of the trajectories, they accelerate, make a smooth loop and return slowly by another route to the space near (0,0,0). This is the typical stimulus evoked behavior of a mono-excitable dynamical system (Huys et al., 2015). The trajectories did not show any sign of equilibrium points outside the (0,0,0).

In addition to these 2 zones the cortex was subdivided into 5 other zones, shown in the colored inserts (Figure 2C). These 5 zones comprise the cortex that did *not* show stimulus related retinotopic localized spiking. For example, the stationary bar did not lead to continuous spiking outside the red zones (Movies 1, 2). The trajectories of the dVSD(t)/dt systematically showed stimulus evoked dynamics in all zones no matter whether the zone mapped the stimulus or not (Figures 2B–D).

The evoked parts of the trajectories were significantly different from the trajectories in the pre-stimulus period. (False discovery rate of 0.05; Methods) (Figure 2). We then tested whether the trajectories from the zone mapping the CFOV in the population of animals (*n* = 8) distinguished the 5 scenes shown in Figure 2 (Methods). There were no significant differences in the evoked part of the trajectories between the scenes with objects moving from the center of field of view, (FDR at 0.05, paired comparison). The scene with the stationary object could be distinguished from scenes with the object moving from the CFOV in the interval 420–460 ms (i.e., after the OFF response). Scenes with a bar moving up or down from the peripheral field of view could be distinguished from 470 to 520 ms post-stimulus (FDR at 0.05 for both conditions). The temporal dynamics of dVSD(t)/dt thus could not differentiate the visual scenes in all cases.

### Space-time sequences of NEX and NINH in areas 17, 18, 19, and 21

**Figure 3:**
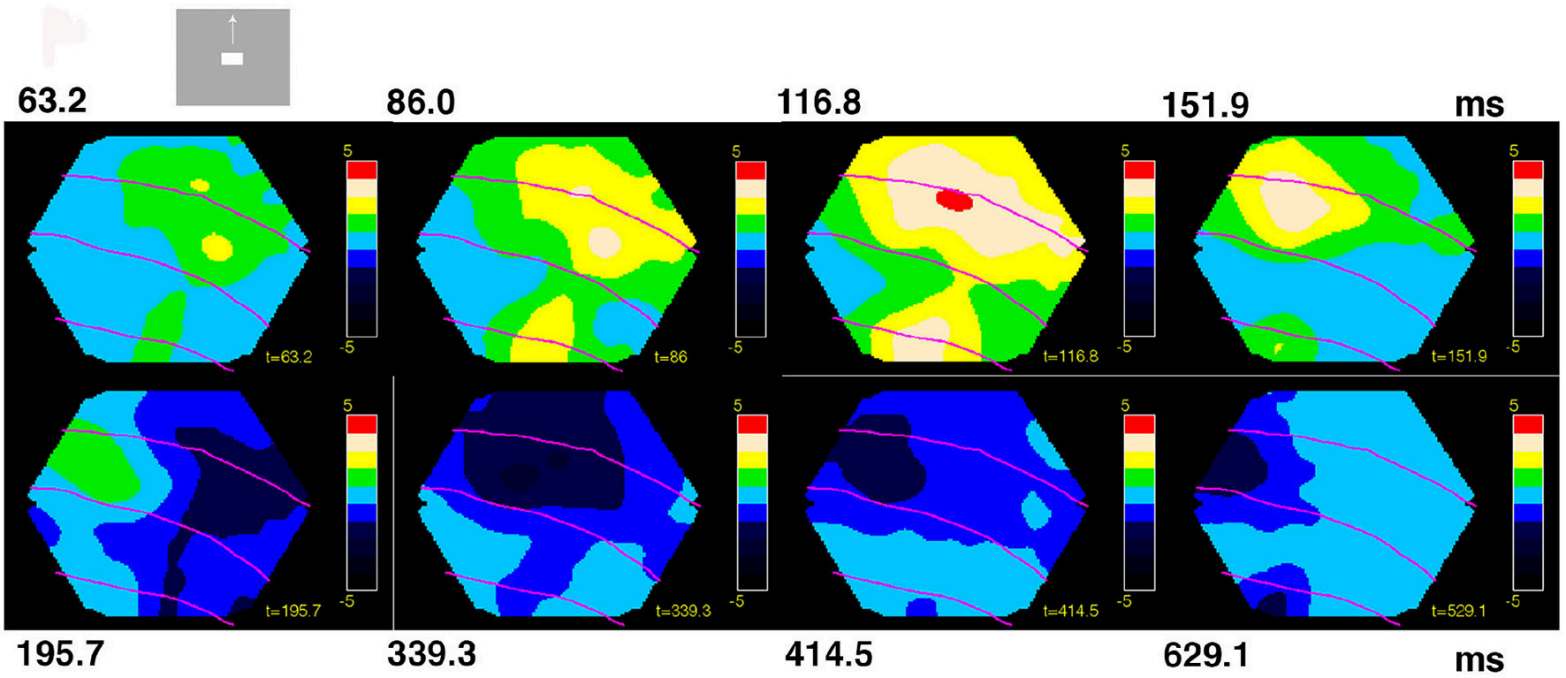
**Space-time dynamics of NEX and NINH to a visual scene with bar moving up from the CFOV**. Non-statistically significant cortex is light blue. Statistically significant NINH dark blue to black scale (FDR at 0.05); statistically significant NEX colors from green to red (FDR at 0.05). Maximum scale values 5 10^−6^. The time after the stimulus start is shown in ms on top and below. Space-time dynamics associated with scene in which a bar moves upwards from the CFOV in animal 2. Non-significant parts, amplitudes from −5 10^−7^ to 5 10^−7^. The sequence of events: The NEX appears and spreads in all directions into the adjacent cortex from the two CFOV mapping sites (8/8). Then an offshoot of the NEX in 19/21 moves toward area 17/18 (7/7) and the NEX at 17/18 moves left (8/8). The NEX reached its maximal amplitude in areas 17/18 (7/8) and in areas 19/21 (5/7). The NEX diminishes and the NINH took over almost simultaneously in areas 17/18 and 19/21 (7/7). The NINH spreads in all directions gaining amplitude (7/7) into all exposed areas (7/8). The NINH shrinks and moves pursuing the earlier NEX in both areas 17/18 (7/8) and areas 19/21 (6/6).

Next we examined the space-time evolution of the statistically significant changes in the balance between excitation and inhibition in the cortical network, i.e., the NEX and NINH space-time changes in anatomical space in each of the 8 animals (FDR at 0.05; Methods). The NEX and NINH were defined as significant increases and decreases above the pre-stimulus, spontaneous state fluctuations in dVSD(t)/dt for each animal (Methods). The appearance of the visual scenes changed the balance into a series of NEX and NINH evolutions in space-time engaging most of the exposed cortex (Figure 3). Identical scenes evoked nearly identical space-time sequences of NEX and NINH in the animals.

For the scenes in which the object appeared in the CFOV and moved either up or down, the statically significant NEX and NINH typical space-time events are summarized by a representative animal in Figure 3 (see also Movies 4, 5 which show the dynamics in anatomical space much better than any figure can convey). The legend to Figure 3 gives the proportion of animals having a particular statistically significant space-time event. The times, after stimulus onset, when these changes of NEX and NINH occurred varied among the animals by up to 70 ms, but the sequence of events was consistent in space-time (Figure 3, Movies 4, 5).

**Figure 4:**
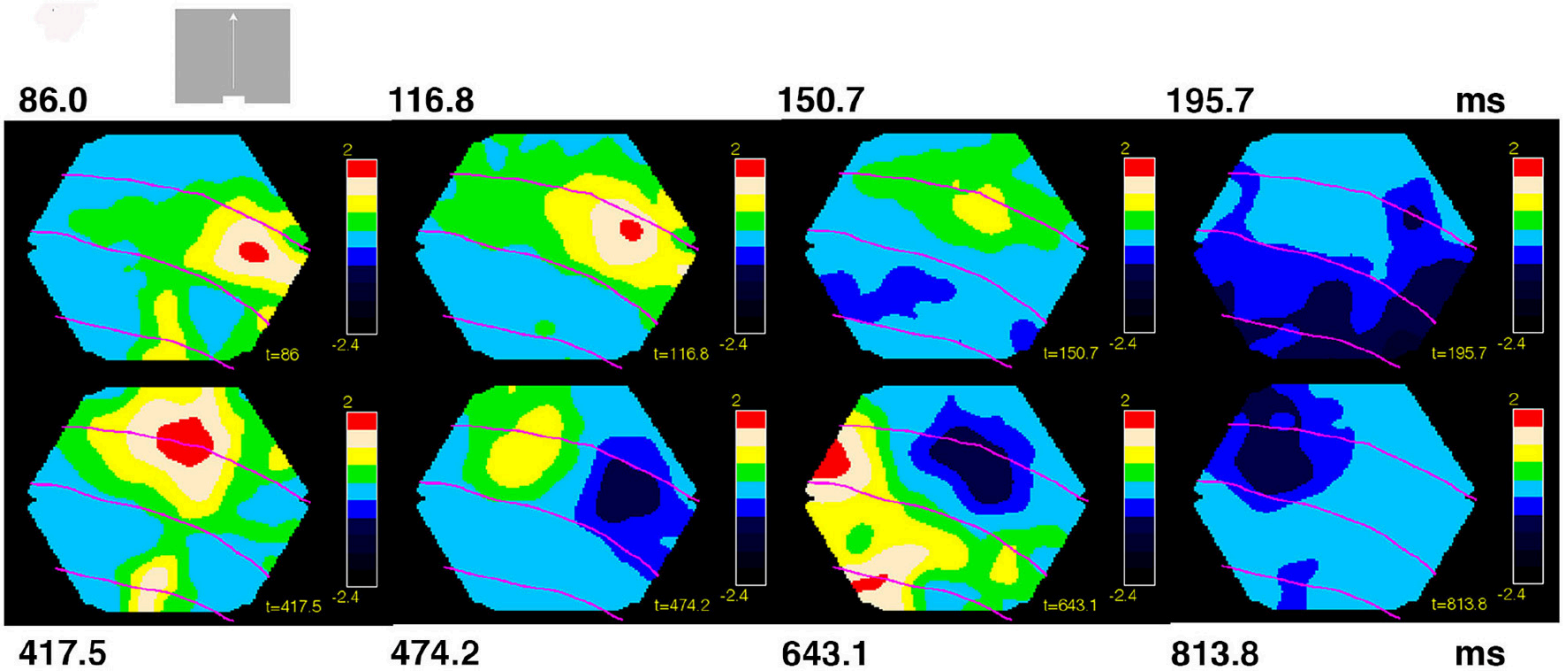
**Space-time dynamics of NEX and NINH for bar moving upwards from peripheral field of view**. One animal shown. Non-significant parts, amplitudes from (−1 to 0.95) 10^−6^. As the bars started 10.5°. From the CFOV, the cortex that was initially active was outside the network monitored by the photodiode array in the majority of the animals. Statistically significant NEX first appeared in area 19/21 (5/7) and in 2/7 animals in area 17/18. The NEX then spreads and an offshoot of the NEX in areas 19/21 moves to the area of 17/18 NEX (7/8). The NEX in areas 17/18 becomes further elongated in the cortical direction of the bar motion (7/7). Then the NEXes in areas 17/18 and 19/21 almost simultaneously attained maximal amplitude (7/7; not shown). NEXes subsequently decrease and switch to NINH almost simultaneously in areas 17/18 and 19/21 (5/8) after which the NINH spreads. In the other 3 animals the NEX was replaced by the balanced state for a while. A second NEX appears in areas 17/18 (7/7) and 19/21 (6/6) spreading from the cortex mapping the CFOV. In 5/7 animals, the NEX decreased and the exposed cortex went into a balanced NEX-NIMH state (shown), in 3 animals, a third NEX appeared immediately and in 5/8 after a little while moving away from the CFOV mapping cortex areas 17/18 (7/7) and areas 19/21 (7/7). Behind the moving NEX a similar moving NINH appeared spreading in all directions. In 7/8 animals the moving NEX and following NINH disappeared from monitoring photodiodes and left the cortex in a balanced NEX/NINH state.

The variability in the timing of the NEX and NINH became even more pronounced in the conditions in which the scenes started with a bar moving from the peripheral field of view. This is characteristic for a dynamical system. The sequence of space-time dynamical events was consistent in the animals also in these conditions, although minor variations could appear in the later part of the space-time evolution (Figure 4). NEX always preceded the first NINH. The NEXs and NINHs had similar space-time dynamics in anatomical space; the NINH space-time dynamics roughly replicated that of the preceding NEX (Figures 3, 4, Movies 4–7).

Neither the NEX nor the NINH evolved to a state with motion of constant amplitude and velocity over the network. In contrast, the balance between excitation and inhibition changed throughout the whole space-time evolution. The changes in the balance engaged large populations of neurons in all animals. (Movies 1–7). The return to small amplitude baseline fluctuations of the excitation-inhibition balance may take 600–700 ms (Movies 4–7).

### Space-time dynamics, but not temporal dynamics alone, distinguish the visual scenes

Figure 1 showed that the mean VSD(t) and the mean dVSD(t)/dt of the population of neurons mapping the CFOV do not distinguish the visual scenes with a bar moving upwards from a bar moving downward from the CFOV. Neither do the temporal course of the dVSD(t)/dt distinguish a stationary bar flashed in the CFOV from the bars moving from the CFOV until the stationary bar disappears and elicits an OFF response (Figures 1, 2). Averaging spatially over the CFOV mapping population or any other neuron population removes the spatio-temporal dynamics.

In contrast, just by looking at the space-time evolution of the balance between excitation and inhibition over the four visual areas, one can distinguish the five scenes (Figures 1B, 3, 4; Movies 1–7). Similarly, just by looking at the space-time evolution of the population membrane potentials, i.e., the VSD(t) caused by stationary and moving objects, over the same four areas, one can distinguish such simple visual scenes in carnivores and primates (Harvey et al., 2009; Ayzenshtat et al., 2010; Harvey and Roland, 2013). Figures 3, 4 show how, when, and where large populations of neurons change their balance between excitation and inhibition or change the membrane potentials at the network scale. However, they do not show how the brain reduces this multidimensional evolution (as many dimensions as points of the network measured) to lower dimension solutions characteristic for a single scene- or if such a reduction is at all possible. For this reason, we examined the space-time dynamics in multidimensional state space. This is in a state space that had as many dimensions as the number of channels in our photodiode camera, namely 464.

**Figure 5:**
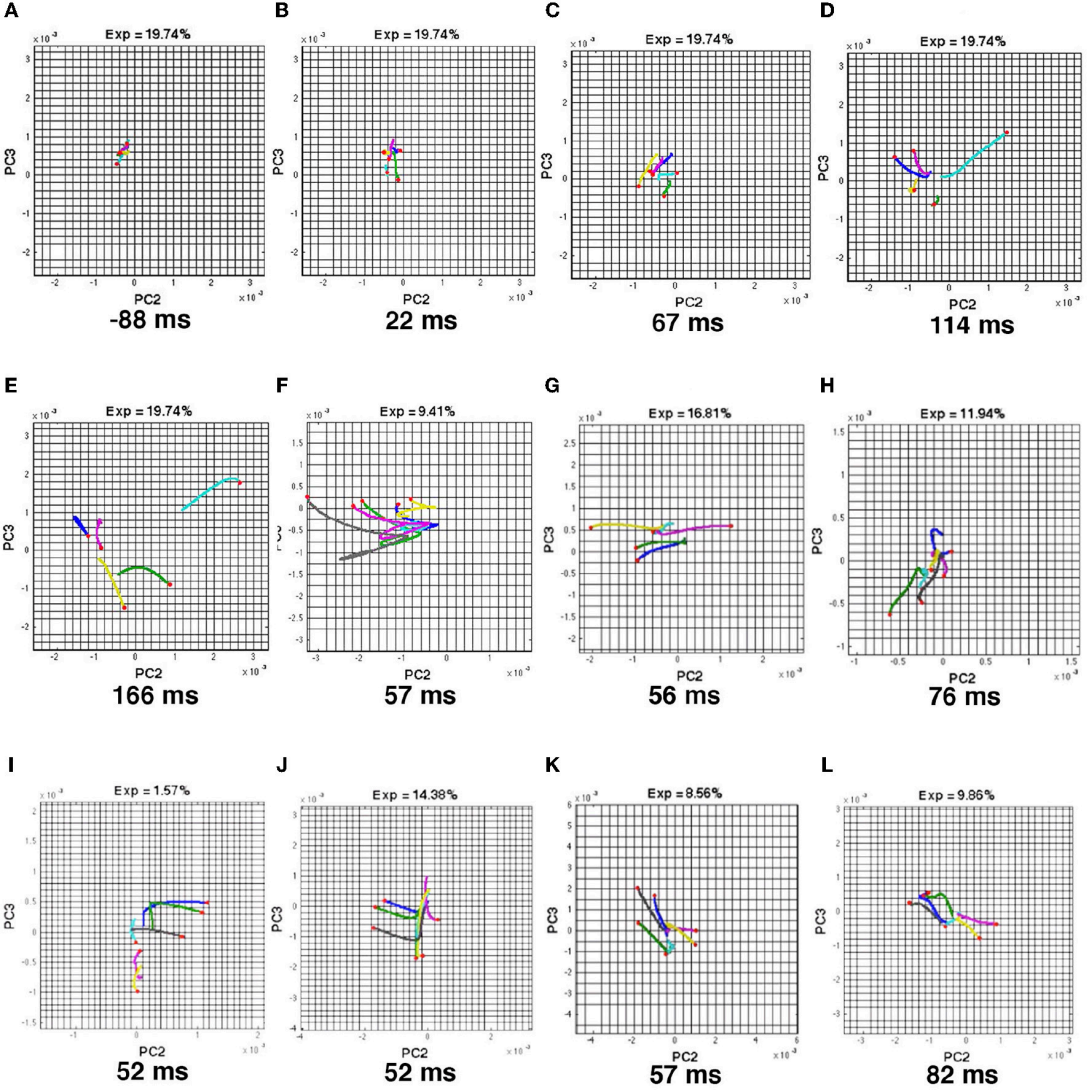
**Space-time evolution of VSD(t) in areas 17, 18, 19, 21 produce flow on low dimension manifold**. State space projection of principal components 2 and 3 of the 464 *spatial* dimension state space (Methods) of all five visual scenes. Each condition's trajectory has a “head” marking its position in state space at the time of the snapshot marked in ms, and a “tail” of its previous 25 ms course. Condition codes: (blue) bar moving down from CFOV, (green) bar moving up from CFOV, (turquoise) bar moving down from periphery, (lilac) bar moving up from periphery, (yellow) stationary bar **(A,E)** Snapshots showing evolution from chaotic-like trajectories to accelerating diverging trajectories (Animal 2). **(F–L)** moment at which the trajectories diverge in animals 1, 3, 4, 5, 6, 7, 8 respectively. For the true dynamics see Movies 8–14.

We calculated the *spatial* PCA (Methods) of the VSD(t) in 464 localizations of the visual cortical network and plotted the first four components. The first 4 components explained 92–99.9% of the total variance. The state-space made of the first 3 principal components explained between 92 and 99% of the space-time variance in the VSD(t) (Movies 8–14). The animals in Movies 10–14 had an extra condition in which two bars moved toward each other (Harvey and Roland, 2013). In state space, the VSD(t) caused by one visual scene produced one trajectory. The trajectories of the VSD(t)s of all scenes first evolved in a chaotic like manner in a small part of the state space throughout the pre-stimulus period and into the post-stimulus period (Figure 5, Movies 8–14). Within 47–82 ms (full range), the trajectories accelerated, diverged, and continued to diverge in state space. This was especially clearly seen in the projection of principal components 2 and 3 (Movies 8–14). This result was consistent in all 8 animals (Figure 5). The time the individual trajectories left the chaotic like state in small part of state space where this behavior took place depended on the time the first statistically significant NEX appeared in the monitored cortex. This delay ranged from −4 to 44 ms (mean 14.0 ms ± 9.0 ms standard deviation). The longer delays seemed related to the time it took for the NEX to spread in the four visual areas.

This means that the evolution of the VSD(t) in all parts of the exposed network first evolved in a chaotic like manner by spatial cooperative activity, similarly to the temporal dynamics in Figure 2. Soon after the upper cortical layers became net excited and the VSD(t) thus increased and this increase spread in the network reducing the complexity of the network membrane potential changes to roughly a 3-dimensional evolution at 47–88 ms progressing in time uniquely for each of the 5 or 6 visual scenes (Movies 8–14).

### Space-time evolution of spiking in areas 17 and 18

We had 82 electrode penetrations in areas 17 and 18. In Movies 1–3 the reconstructed position of each site is mapped on the hexagonal photodiode array monitoring the visual areas. Depending on how many of the 50 trials that showed evoked spiking in a certain ms (see Methods for definition of evoked spiking), the color changed from light blue to red. Movies 1–3 show initial spreading of the evoked spiking in the network up to 100 ms. For the stationary object, the evoked spiking outside the retinotopic mapping of the object in areas 17 and 18 ceased in the granular layer, but continued albeit weakly in the supragranular layers (Movie 2). For the scenes with moving objects, there was some spiking ahead of the retinotopic evoked spiking at 120–200 ms, characteristic for each scene (as described by Harvey et al., 2009) (Movie 3). Thereafter there was a more ordered progression of retinotopic spiking (Movie 3) (additional movies for spiking to the other scenes at https://www.dropbox.com/sh/o6qtfrhmt5jwev2/AABbxksHtkd0UxTlFCrA_b0ca?dl=0). However, only the spikes from a single penetration were obtained simultaneously, therefore it made no sense to perform a spatial PCA on these reconstructed data (Methods).

If one compares the space-time evolution of the evoked spiking (Movies 1–3) with the space-time evolution of dVSD(t)/dt and VSD(t) (Figures 1–4, Movies 4–7) the VSD(t) changes and the changes of excitation-inhibition balance take up a larger territory of the network in areas 17 and 18. Furthermore, it is obvious that the spiking, even in the upper cortical layers from ~125 to 300 ms and in periods thereafter, continues seemingly unaffected by the NINH regimes.

### Delayed balance

So far our results unambiguously showed that the smaller fluctuations in the balance after the impact of the visual scenes is replaced by a large amplitude NEX that after some 100 150 ms switches to a NINH (Figures 1, 3, 4, Movies 4–7). The network of the four visual areas could still be balanced, in the sense that inhibition sooner or later would bring it back to a stable state when the impact of the scenes cease (Huys et al., 2015).

We examined the local amount of NEX and NINH (*p* < 0.05, Methods) in the four areas by integrating the NEX over the whole post-stimulus period 0–800 ms and then dividing by the number of samples and then repeating this for the NINH (Methods).

**Figure 6:**
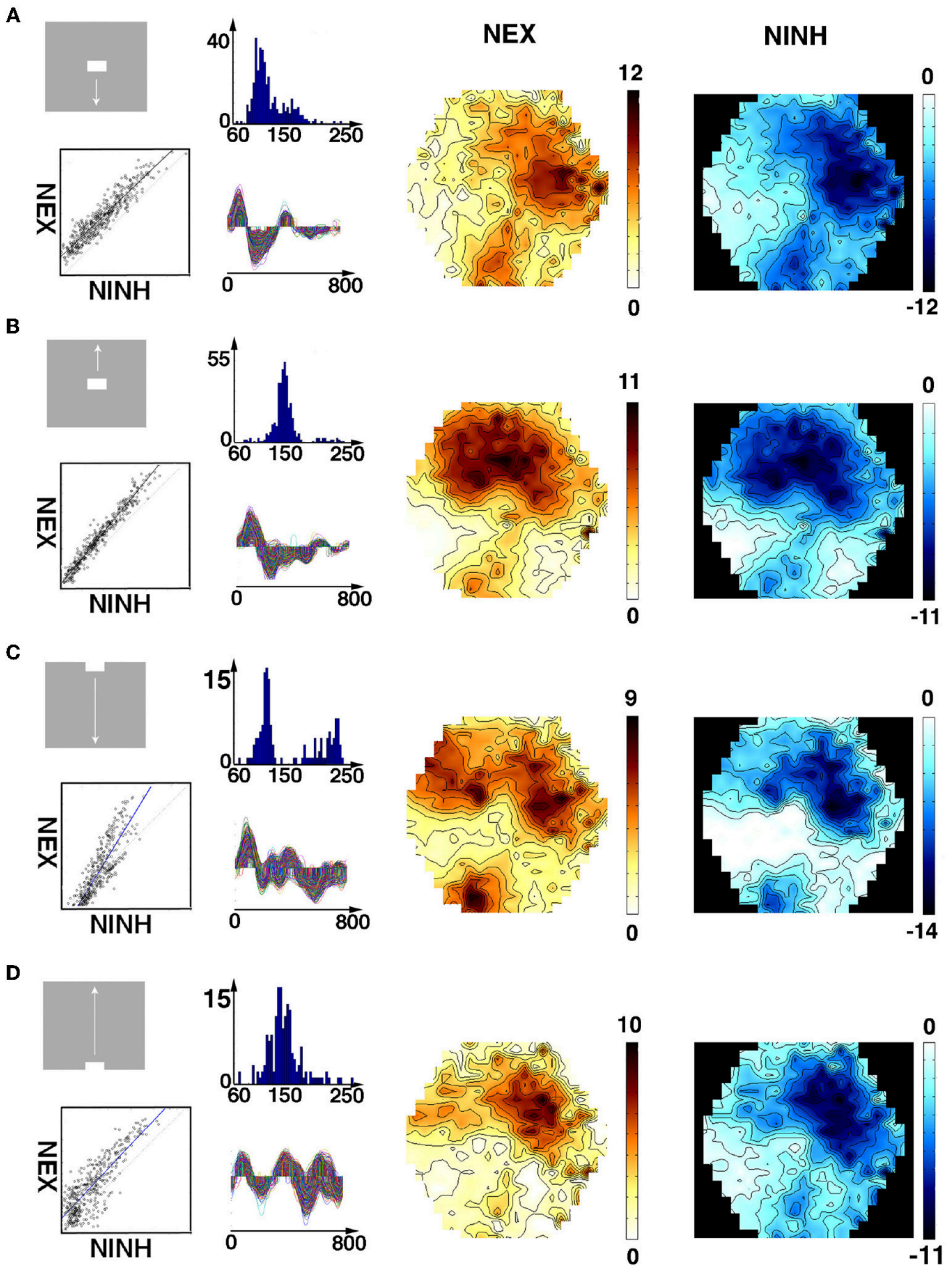
**Spatial distribution and correlation of average NEX and NINH**. First column: stimuli and correlations of average NEX and NINH at the cortical locations having (statistically significant) NEX and NINH. Second column: Top images: distributions of the delay between maximum NEX and the NINH minimum for each cortical location. Bottom: time-varying significant (*p* < 0.05) NEX and NINH from each of the locations in the four visual areas. X-axis ms. Third column: spatial distribution of average NEX in the 4 visual areas. Last column: spatial distribution of NINH in the 4 visual areas. Scale values should be multiplied with 10^−7^. The average NINH at each location is proportional to the average NEX. Across animals and conditions, the slope of the correlation was not significantly different from 1. **(A)** Object moving down from CFOV, one animal. **(B)** Object moving up from CFOV, one animal. **(C)** Object moving down from peripheral FOV, one animal. **(D)** Object moving up from peripheral FOV, one animal.

The integrated NEX matched the integrated NINH in distribution and amount in all 4 areas (Figure 6). The correlations between the average integrated NEX and average integrated NINH for all cortical locations in all animals and all 4 conditions of moving bars (*n* = 32) were all highly significant (F-statistics *p* < 10^−10^). The average delays between the maximum NEX and the minimum of the NINH (*n* = 16; Methods) were for the two conditions with the bar starting at the CFOV 118 ± 25 ms (mean ± stand. deviation). For the two conditions with the bar starting in the peripheral field of view 138 ± 26 ms and for the stationary square condition 125 ± 37 ms. The average slope of the regression was 1.00 ± 0.31 (mean ± stand. deviation) indicating that on the average, the amount of NEX became balanced by an equal amount NINH. These results were largely independent of the duration of the period over which the NEX and NINHs were integrated. For example, reducing the period to be from 0 to 400 ms and even from 0 to 210 ms after stimulus onset did not affect the strong correlation between NEX and NINH. The figures of average integrated NEXs, NINHs and their spatial correlations in all animals and conditions are available at https://www.dropbox.com/sh/j6bs1eegvh14fth/AAB-HYQPhallfh-wH9SDe4nPa?dl=0.

These results showed that there was no instantaneous or fast, tight balance between NEX and NINH after the perturbation by the stimuli. Instead, at any spot in the participating cortical network, the net-excitation was after a delay of ~125 ms balanced by an equal amount of net-inhibition. This also implied that the NEX and NINH occupied identical network territory (Figure 6).

### Propagation speeds of NEX and NINH

**Figure 7:**
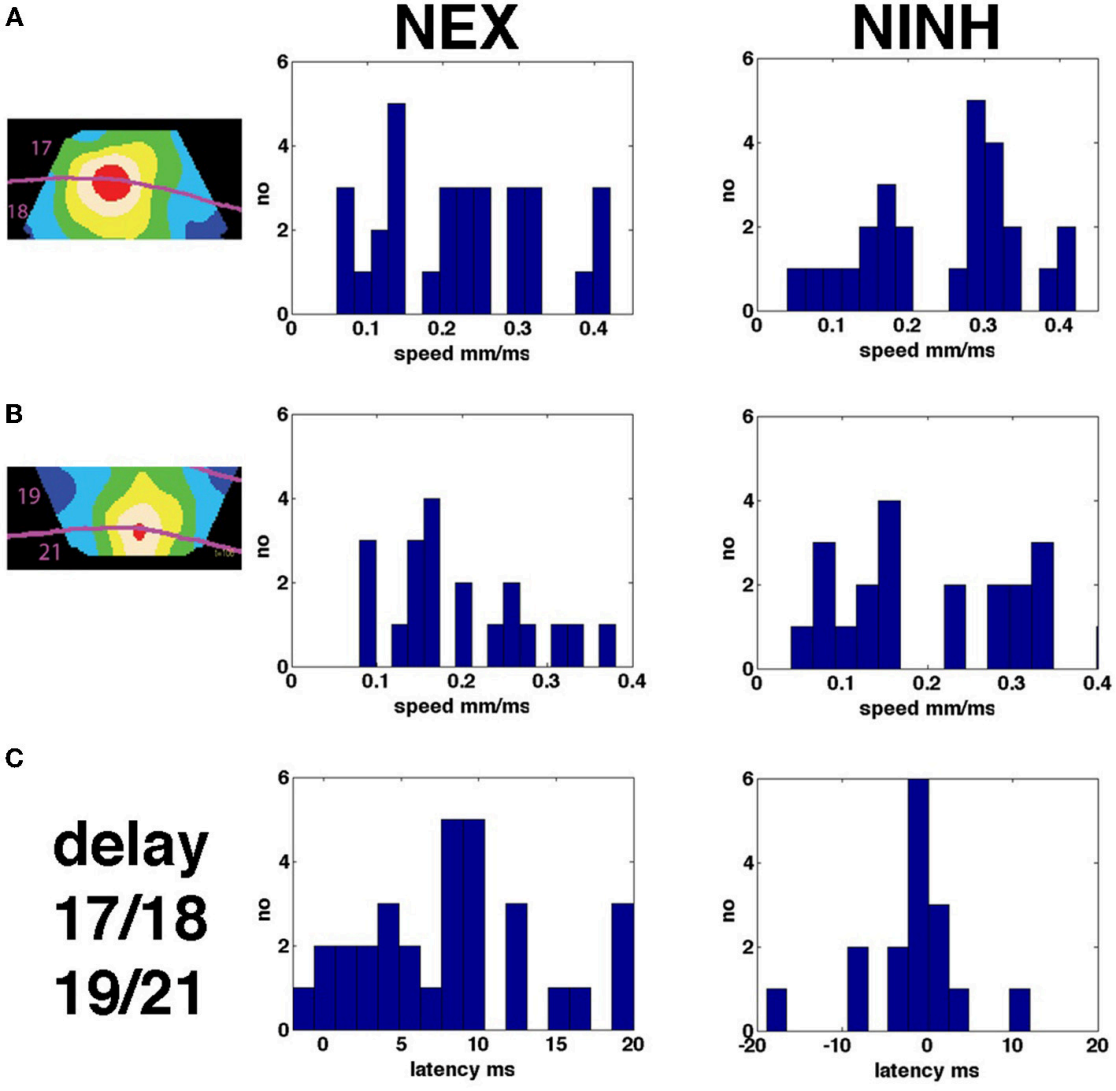
**Speeds of NEX and NINH and delays between areas. (A)** Distribution of speeds of propagation in areas 17 and 18 of the first NEX and NINH for all four scenes with moving objects **(B)** Same but for areas 19 and 21. **(C)** Distributions of minimum latencies of the first NEX and NINH in ms between areas 17/18 and area 19/21.

There were several forms of propagation of the NEX and NINH in the cortical network. First, the VSD(t) spreads out from the retinotopic mapping site in area 17 when a visual object is flashed in the FOV (Grinvald et al., 1994; Roland et al., 2006). Then the NEX became oriented in the direction mapping the trajectory of the object and then the NEX branched out from areas 19/21 moving toward the NEX in areas 17/18. Examination of these dynamics show that they were partly superimposed, i.e., after a while it is not possible to describe the NEX of the membranes as a single “wave” having one direction (Figures 3, 4, Movies 4–7). As seen in Figure 7 there was a relatively large range of speeds of both NEX and NINH spanning from 0.08 to 0.4 mm ms^−1^. The distribution of speeds was similar in areas 17/18 and areas 19/21. The mean speed ± square root of the variance was 0.23 ± 0.10 mm ms^−1^ for the initial NEX and 0.21 ± 0.08 mm ms^−1^ for the first NINH in areas 17/18 with no statistical difference between NEX and NINH (*p* > 0.2; *t*-test). In areas 19/21 the speeds were 0.20 ± 0 .08 mm ms^−1^ for the first NEX and 0.22 ± 0.12 mm ms^−1^ for the first NINH with no statistical significance (*p* > 0.1, *t*-test). In summary there were no differences between the speeds of the first NEX and the first NINH propagation and no difference in speeds between the primary visual areas 17/18 and the higher areas 19/21. The progression speed of the second NEX was (*n* = 10) 0.25 ± 0.10 mm ms^−1^ and the second NINH (*n* = 16) 0.19 ± 0.09 mm ms^−1^.

### Phase delays

**Figure 8:**
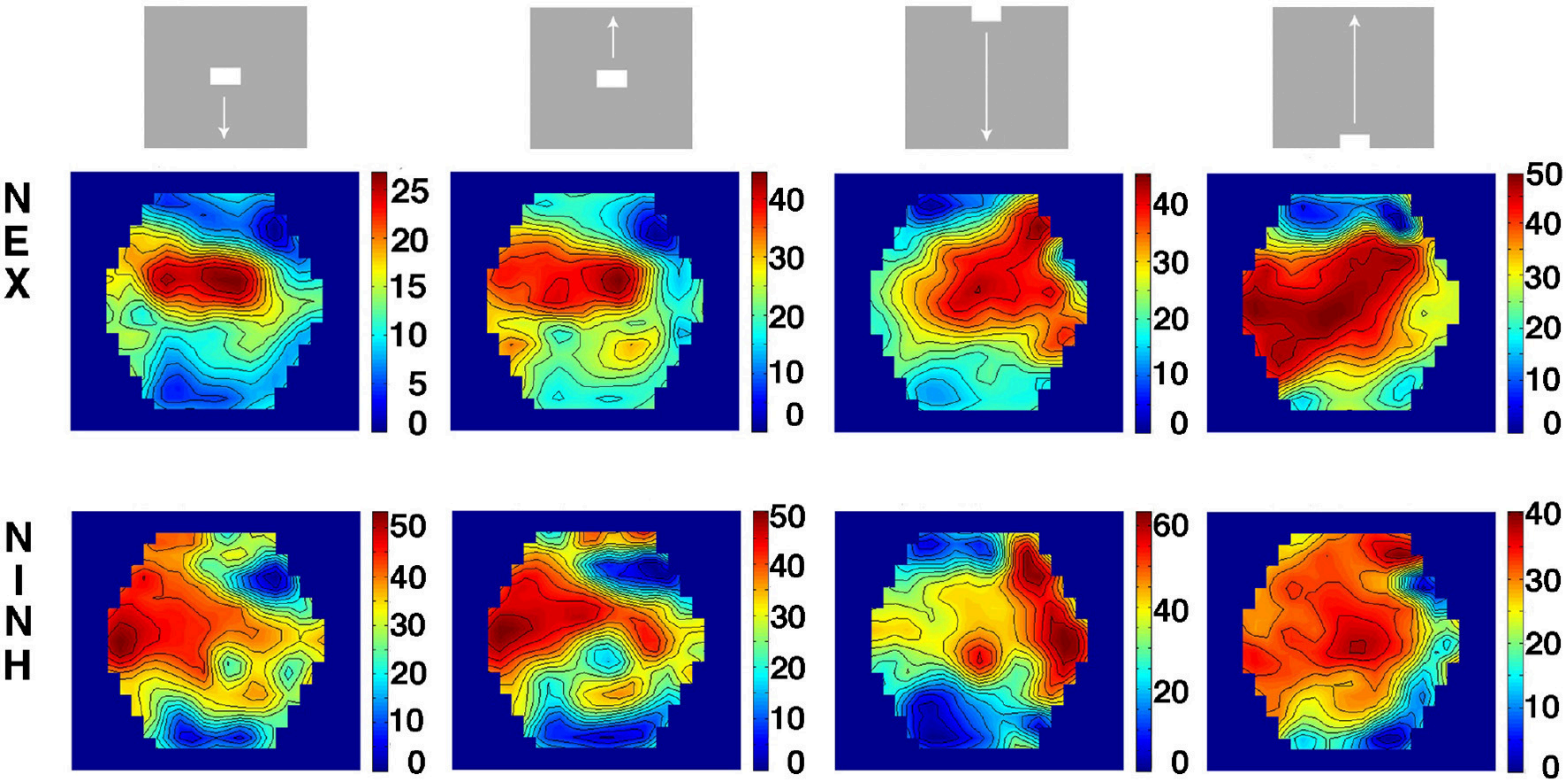
**Phase latency maps of first NEX and NINH in four scenes**. The Hilbert plots (Methods) show the phase latencies of the first NEX and NINH in ms between the start location in cortex (having the minimum latency) and the remaining parts of the cortex. Note the 5–10 ms delays between area 17/18 (top of the hexagon) and areas 19/21 (bottom of hexagon) for NEX start. Animal 1.

When the objects moved along on the display screen, first the neurons in areas 17 and 18 spiked and went into NEX, then the neurons in areas 19 and 21 spiked and went into NEX. Thus, the NEX will appear in several versions in several visual areas, each delayed more and more compared to the primary visual cortex. We asked whether there existed a mechanism that brought the membrane potentials into phase. In order to calculate the phase differences in the cortex, we Hilbert transformed the data files (Müller et al., 2014; Methods). Calculated over a time interval of 100 ms, the Hilbert transformed data gives a single image of the cortex showing the phase latencies in ms (Figure 8). The NEX delay between areas 17/18 and areas 19/21 in all animals and conditions is shown in Figure 7 and was 8.35 ± 5.74 ms (mean ± square root of variance) and significantly greater than 0 (*p* < 0.00002; *n* = 30). The NINH delay between area 17/18 and areas 19/21 was not significantly different from 0 (*p* > 0.9; *n* = 23) in mean −4.30 ± 9.92 ms. This indicated that when the NINH starts, the phase differences were not obvious. To further illustrate this we spatially aligned the VSD(t) measurements of animals with roughly matching cytoarchitectural borders by simple affine transformations (Methods) to show the space-time evolution of the normalized nVSD(t) across animals. Normalizing all pixels to their max being 1, removes the amplitude differences but maintains their phase relations. Movie 15 (*n* = 6) shows that, for the bar moving down from the peripheral field of view, the nVSD(t) in areas 19/21 leads that of areas 17/18 from 60 ms up to 116 ms and thereafter the area 17/18 nVSD(t) catches up to nullify the phase differences at 160 ms and onwards.

## Discussion

We presented the following main results. The appearance of the visual scenes drove the network away from a more tightly balanced state into space-time sequences of expansive NEXs and NINHs. NEX and NINH propagated through the network with similar speeds. Locally in the network, the amount of NINH was strongly correlated with the amount of NEX appearing 125 ms earlier. This implied that the NIMH matched the NEX spatially and dynamically, albeit with a delay. The temporal dynamics of the NEX and NINH could not distinguish the visual scenes in all cases. The space-time dynamics in all parts of the exposed network evolved early to reduce the complexity of the network membrane potential changes to roughly a 3-dimensional evolution progressing in time uniquely for each of the visual scenes.

### Temporal dynamics

Our *in vivo* measurements and data treatment of the VSD_i_(t) impose some constraints on the interpretation of the results. First, we averaged the single trials to get the VSD(t). Second we applied a temporal filter on the VSD(t) to exclude high frequency (photon) fluctuations. Third, the VSD_i_(t), at each cortical point of measurement, stems from dendrites and axons belonging to tens of thousands of neurons (Roland, 2010). This implies that individual fluctuations of the sum of membrane currents have been suppressed, and therefore we can only characterize the average behavior of the network. Fourth, because the cell bodies only make up a minute proportion of the total membrane surfaces of neurons (Fiala and Harris, 1999), the NEX and NINH may not agree with somatic patch clamp data (Williams and Mitchell, 2008). Fifth, the dye signal is proportional to the surface of the stained membranes, thus signaling mainly the membrane potential changes in large populations of dendrites and un-myelinated axons in the upper layers of the network. This means that the membrane compartment providing the NEX and NINH is a mixture of pre- and post-synaptic elements. Sixth, in *in vitro* experiments, 1% isoflurane concentration increase a K^+^ leak current modestly and linearly in thalamic neurons (Ries and Puil, 1999), which could depress the spiking driving the NEX. In another *in vitro* experiment the dye RH 1691 enhanced the GABA-A response at concentrations used in our experiments (Mennerick et al., 2010). Thus, the dVSD(t)/dt results may be slightly biased toward NINH. However, a recent modeling study show that these inhibitory effects might be negligible (Chemla and Chavane, 2016). In addition, inhibition in anesthetized mice (isofluran and chlorproxtiene) was weaker than in awake mice (Haider et al., 2013). Finally, at each point of measurement, the NEX and NINH only report relative changes in the sum of the ion currents in the membrane compartment. Consequently, the NINH cannot distinguish decreases in the sum of inward currents from increases in the sum of outward currents and the NEX does not distinguish increases in inward currents from decreases in outward currents.

These limitations, however, cannot effect the results that the presentations of visual scenes with moving objects lead to longer, 70–200 ms lasting episodes of NEX and NINH; nor that the amount of NEX locally in the four visual areas subsequently is matched by an equal amount of NINH. The impact of the afferent potentials switched the balance in the visual cortex from a tight temporal balance to a delayed balance. The period of net-excitation permits active spiking to propagate through the network. When the network is perturbed by stimuli, its connectivity and the behavior of the inhibitory neurons allow larger deviations from the spontaneous state. When the visual scenes disappear, the delayed balance implies that the network will return to the regime of smaller fluctuations from the baseline, although slowly (Figures 3, 4; Movies 4–7).

Our results complement studies of balanced excitation and inhibition performed on single cell bodies. Although our results are incompatible with a tight temporal balance of excitatory and inhibitory currents after the impact of a change in the visual scene and although our cortical VSD(t) is practically blind to what happens at the somata of the neurons, the result that there is a 125 ms delay in the cortical network between maximal net excitatory and maximal net inhibitory currents is similar to recordings from the cell body. In a subset of earlier single neuron patch clamp findings in lightly anesthetized cats and ferrets similar or even longer delays were found between excitatory and inhibitory conductances (Anderson et al., 2000; Martinez et al., 2002; Monier et al., 2003; Priebe and Ferster, 2005; Tan et al., 2011; Baudot et al., 2013). However, the delay between excitation and inhibition may depend on animal order (Tan et al., 2011). For carnivores it may also depend on the type of visual stimulus, e.g., drifting grating, object or a natural scene (Anderson et al., 2000; Martinez et al., 2002; Monier et al., 2003; Priebe and Ferster, 2005; Tan et al., 2011; Baudot et al., 2013). Natural scenes might evoke shorter delays than those we observed (Baudot et al., 2013). Our results are compatible with observations that the spiking of excitatory and inhibitory neurons matches over longer time scales (seconds) (Dehghani et al., 2016). Our result gives the overall delay in dendrites, un-myelinated axon terminals and cell bodies belonging to an estimated 400,000 neurons, that the cell body measurements cannot predict. As seen from Figure 6 (second column) the delay varied locally from 60 to 250 ms. This is what would be expected from large network with space-time dynamics. Our overall result does not exclude shorter or longer delays in selected single neurons.

Onat et al. (2011a) computed the average time courses for a drifting grating and natural scenes and estimated the decay VSD signal, VSD(t), by a straight line. “The processing of different gratings showed a strong adaptational decay of net excitation levels. For gratings, its negative slope was equal to −48%/s (99% CI = [−59%/s, 35%/s]), meaning that after only 1 s of presentation time amplitudes declined to half of the peak activity levels. During stimulation with natural movies, the mean adaptational decay was −24%/s (99% CI = [−44%/s, −5%/s], and the median slope was found to be only marginally different from zero (sign test, *P* = 0.065).” From which they drew the conclusion: “Thus, drifting oriented gratings are processed in a non-stationary regime and in contrast, natural movies processing is generally characterized by overall stationary balance between excitation and inhibition” (Onat et al., 2011a). Neither Eriksson et al. (2008), Harvey et al. (2009), Harvey and Roland (2013) nor we (Figures 1B, 6) could fine any time segments over the first 800 ms in which the temporal course of the dVSD(t)/dt of the post-stimulus period to stationary and moving stimuli was approximately linear. This indicates that the time course of the balance between excitation and inhibition in response to these stimuli most likely is dynamic, i.e., changing continuously.

The time course of the inhibition may depend on the duration of the stimuli. The shorter the stimulus, the deeper the inhibition elicited by the stimulus offset (Eriksson et al., 2008). When two bars move toward each other in the field of view, the local balance between excitation and inhibition turn to inhibition in advance of their mutual occlusion. For the time interval of occlusion, the spiking locally at the retinotopical spot in primary visual cortex becomes tuned to that representing one single bar (Harvey and Roland, 2013). This might illustrate a property of the local network to modulate the balance for fine-tuning of local visual details.

It is often claimed that the cerebral cortex is similar to a balanced neuronal network.

The visual cortex is continuously spiking *in vivo*, even when it is not driven by external stimuli (Hubel and Wiesel, 1959). The spiking in this spontaneous state is slow and irregular (chaotic like) associated with moderate amplitude fluctuations of the membrane currents indicating a relatively tight balance of excitation-inhibition (Haider et al., 2006; Monier et al., 2008; Huys et al., 2015; Figure 1A). This however does not necessarily mean that the cerebral cortex behaves like a balanced neuronal network. Even in very large balanced neuronal networks with many biologically relevant details, the spiking rapidly dies out unless it is driven by an external (artificial) input (Izhikevich and Edelman, 2008; Kumar et al., 2008; Markram et al., 2015). This holds also for the classical balanced neuronal network that swift and nearly linearly responds to an increase in the external drive with a new equilibrium between excitatory and inhibitory currents (van Vreeswijk and Sompolinsky, 1996), which is in contrast to our results (Figures 1–6, Movies 8–14).

### Space-time dynamics in the cortical network

We described the space-time dynamics of the membrane potential changes in two different spaces: the topological (anatomical) space made of the cortical network of synaptic connected neurons (Figures 1, 3, 4, 8; Movies 1–7, 15) and the (abstract) state space depicted as orthogonal principal component projections (Figures 2, 5, Movies 8–14). In the cortical network anatomical space one can see the spatial evolution of NEX and NINH in time and get an intuitive sense of their space-time dynamics.

The visually evoked space-time dynamics of the dVSD(t)/dt started with a NEX in a small zone spreading in all directions, including the direction opposite to that mapping of the moving object. After this followed a NINH with similar space-time dynamics (Figures 3, 4, 8; Movies 4–7). Although the VSD(t) increase appearing after visual scene shifts always spreads laterally in the cortex (reviewed in Roland et al., 2014), we now show that this is composed of NEX dynamics followed by a similar spreading NINH dynamics. The spreading NEX dynamics enable the spiking related to two different objects to interact at a distance and interact with the visual background (Harvey and Roland, 2013).

The speed of NEX spread varied from 0.1 to 0.4 mm ms^−1^ in all four areas from the two cortical sites mapping the retinotopic position in areas 17/18 and 19/21 (Figures 3, 4, 8), indicating that the NEX spread may be fast mono-synaptic from the mapping neurons as well as more slowly poly-synaptic via neurons not mapping the bar in the cortical surround. The poly-synaptic hypothesis is in accordance with the result that there were neurons entering the evoked spiking state, outside the CFOV retinotopic site, for a short instance initially (Movies 1–3). These speeds are also in accordance with the spread of VSD(t) increases observed in primates V1 and V2 by Müller et al. (2014).

Speculatively, the high-speed spread of the NINH into the adjacent cortex could be done mono-synaptically by long axons from inhibitory neurons spiking in the mapping zone (Kisvarday et al., 1997). The slower spreading of NINH could hardly be poly-synaptic, but caused by the disappearance of excitation locally combined with local spiking of inhibitory neurons. At the mapping site, the spiking increased again after 80 ms this might be an increase of spiking mainly by inhibitory neurons, because this spiking increase coincided with the NINH getting deeper (Figure 1). As the excitatory drive now was gone (because the object moved) this would cause the excitation to decrease also locally in the surroundings of the mapping zone where the always spiking inhibitory neurons would take over (Rudolph et al., 2007).

The first NINH covered large regions, including the retinotopic spiking zones related to the object 125 ms after the NEX (Figures 3, 4, Movies 4–7). However, the layer 4 and 2, 3 neurons continued to spike at these subsequent retinotopic sites during the NINH, (Movie 2). Apparently, this spiking was insufficient to drive the network into NEX. This is not so surprising as the VSD-signal reflects membrane potential changes of all membranes (Methods). The finding, though, illustrates that the network around spiking excitatory neurons can be in a different regime. The widespread inhibitory regime might prevent the spread of the evoked spiking in the interval 100–200 ms after the start of the stimuli and hence confine the evoked spiking to the retinotopic zone (Movies 1–3).

The individual variations in appearance of second NEXs and NINHs and third NEXs and NINHs became more pronounced as time after the stimulus onset increased, as expected for a dynamical system. A full description of individual variations should ideally be on a single trial basis, which was not possible for the present data of dVSD(t)/dt.

Some theoretical models replicate essential parts of the VSD(t) space-time dynamics to stationary and moving objects in the primary visual cortex of primates and carnivores. This includes the lateral spreading of the VSD(t) increases and VSD(t) space-time dynamics giving rise to visual illusions (Rangan et al., 2005; Deco and Roland, 2010; Markounikau et al., 2010). Markounikau et al. (2010) show that the VSD(t) most likely is composed to a large extent of inhibition, modeled as the time derivative of the population membrane potentials, which is in accordance with our results (Figure 1). However, none of the models have spontaneous ongoing membrane current fluctuations and hence do not predict the transition from tight to delayed balance. In the Chemla and Chavane's (2016) model different delays between excitatory and inhibitory spiking modulates the shape of the VSD(t) and hence affect also the dVSD(t)/dt. Although many models, including the above mentioned, have a strong local coupling between excitation and inhibition, in accordance with (Figure 6), to our knowledge, this has not lead to predictions of the space-time dynamics of the long delays between NEX and NINH (Figures 3, 4, 6). One model study predicts the NEX propagation from area 19/21 toward areas 17/18 at 80–100 ms (Figures 3, 4; Deco and Roland, 2010). One recent model predicts that excitation and inhibition under certain synaptic regimes can segregate spatially at the mesoscopic (network) scale (Malagarriga et al., 2015). This is in contrast to our finding of a strong local correlation between NEX and NINH in the four visual areas and the results in Figures 3, 4, 6.

When the scene appears at time 0, first the spiking from the lateral geniculate nucleus breaks the tight balance between excitation and inhibition and drives the network into NEX and evoked spiking at the retinotopic spot for the bar (Figures 1, 3, 4; Movies 1, 3). The temporal dynamics of the NEX at this spot of the network does not distinguish a stationary bar from bars moving up or down (Figure 2), neither do the temporal course and the temporal dynamics of the evoked spiking (Movies 1–3; Forsberg et al., 2016). The evoked spiking at this initial spot drives the surrounding network into NEX with similar temporal dynamics engaging the network over nearly all of the exposed cortical surface (Figure 2; Movies 4–7). However, after 40 ms, the driving by the retinal input spikes has moved to an adjacent spot in the network where the previous spreading NEX facilitates the evoked spiking (in accordance with the models of Rangan et al., 2005; Markounikau et al., 2010). At this new spot, the spreading NEX changes to be more specific to the visual scene driving it, at 45–90 ms and onwards (Movies 4–7).

The evolution of the NEX and NINH in state-space is a succinct mathematical description of the collaboration of all parts of the exposed cortical network comprising the four visual areas. The state-space made of the first 3 principal components, i.e., a manifold, is a mathematical object on which the flow of the NEX and NINH for each scene evolves. The evolution of the specific space-time dynamics starts with the divergence of the trajectories at 30–90 ms showing the cooperative membrane potential changes of the total network. The departure from the chaotic like behavior and divergence of the trajectories coincided with the NEX spreading to cover most of the exposed cortex. The trajectories continued to diverge over the next 50 ms when the net inhibitory regime takes over at 110–120 ms (Movies 8–14). It is at this point of time the evoked spiking becomes more clearly related to current scene (Movies 1–3; Eriksson et al., 2010). The trajectories on the 3-dimensional manifold thus show the space-time dynamics of the membrane potential changes leading up to the 110-120 ms when the scene is likely to be perceived (Thorpe et al., 1996; Chen et al., 2007; Eriksson et al., 2010).

Onat et al. (2011a,b) using a drifting grating as stimulus and principal component analysis showed that some components reflected the wave propagation from the moving gratings, whereas the first 2 components reflected the stationary feature, i.e., the orientation of the grating. If one looks at Movies 8–14, one can see that the first 3 components contribute to the divergent flows over the manifold, although the divergence was slightly larger in the projection of the 2nd and 3rd component. Thus, in our case there was no (clear) separation of space vs. time in the space-time dynamics. In our case, the retinotopic mapping of the moving object moved over the cortical network, so one could think that this was the reason for the discrepancy. However, if one focus on the flow associated with the stationary square (yellow in Movies 8, 9, gray in Movies 10–14) this was also affected by the first 3 components. Another reason for the discrepancy could be that the first 400 ms were discarded from the analysis in the Onat et al. (2011b) study. In contrast, our stimuli and analysis was starting from 0 ms and thus biased toward spatio-temporal transients.

The evolution to low dimensional flow would not have been possible if the network had remained in tight balance, as also seen in Movies 8–14. The spontaneous state is a stable state (Huys et al., 2015; Forsberg et al., 2016). For sensory areas, in general, the tight balance between excitation and inhibition must be broken to allow the evoked spiking and NEX to propagate in the network to engage as many neurons as possible in the dynamics driven by the scene. Even if the neurons are only engaged sub-threshold, this makes the solution of the network robust. The subsequent NINH appearing locally in the network at 100–110 ms, removes excess spiking outside the part of the network engaged in mapping the progress of the bar, and hence probably sharpens and further stabilizes perception (Movies 1–3). It remains to be tested whether the dynamic separation also works in single trials. This question was not possible to answer with our present data.

## Conclusions

We measured the balance between net-excitation, NEX, and net-inhibition, NINH, in the network of dendrites and un-myelinated axons in the upper layers of four visual areas. Our results complement studies of the excitation-inhibition balance in the cell bodies of single neurons. The appearance of visual scenes with moving objects elicited longer, 70–200 ms lasting episodes with larger deviations of NEX and NINH from baseline, and at times, sequences of several NEXs and NINHs, implying that it took some 600–700 ms for the balance to return to the state of small fluctuations. These results are incompatible with a fast, tight balance between excitatory and inhibitory currents and with the behavior of a classic balanced network (van Vreeswijk and Sompolinsky, 1996). Locally in the network, in all four areas, the amount of NINH with a delay matched the amounts of NEX for these episodes. The NEX and NINH thus were also matched spatially in the network, in our case within 800 ms, by local inhibition. Thus, the network should return to the baseline or pre-stimulus state, when the last episode of NINH is finished.

The *temporal* dynamics of the changes in the population membrane potentials, the dVSD(t)/dt, spread to all zones of the visual areas with similar trajectories resembling those of a mono-stable excitable system. In contrast to the temporal dynamics of the dVSD(t)/dt and hence the NEX and NINH, the NINH and NEX space-time dynamics in anatomical space as well as in abstract state space explained how differences evolved to distinguish simple visual scenes. We do not know whether these mechanisms also will explain differences in natural, complex scenes. Space-time dynamics of the changes in the balance between excitation and inhibition evolved at a much larger scale than that used to examine receptive fields changes in single neurons. The space-time dynamics are missed with local probes, and are only captured by a high frequency sampling network approach. The results of the collaboration in this large network were succinctly described as diverging trajectories after 50–88 ms on a low-dimensional (manifold) in state space. The network in the four areas operated with similar local temporal dynamics to the presentation of the visual scenes, whereas its (average) spatio-temporal dynamics evolved to distinguish the scenes.

## Author contributions

PR and MH designed the experiments, MH did the experiments, LB, LF, and PR designed and did the data analysis. PR wrote the final version of the manuscript.

### Conflict of interest statement

The authors declare that the research was conducted in the absence of any commercial or financial relationships that could be construed as a potential conflict of interest.

## Supplementary material

Movie 1 **Evoked spiking in the granular layer to a scene with a stationary bar shown at time 0–250 ms**. The movie starts 200 ms prior to stimulus onset. The electrode penetrations are marked with white circles on the photodiode map. The magenta curve marks the position of the cytoarchtectural border between areas 17 and 18 (Methods), which obscured one channel to the right. The color scale shows how many trials (out of maximum number of trials) that are evoked. Note the evoked spiking outside the zone mapping the CFOV shortly after stimulus onset.

Movie 2 **Evoked spiking in the supragranular layers to a scene with a stationary bar shown at time 0–250 ms**. Conventions as in Movie 1.

Movie 3 **Evoked spiking in the granular layer to a scene with a bar moving up from the CFOV**.

Movie 4 **Space-time dynamics of statistically significant NEX and NINH (FDR methods < 0.05) in one animal to a scene with a bar moving down from the CFOV**. The color scale values should be multiplied with 10^−6^. Magenta curves show cytoarchitectural borders between areas. The time in ms after the start is shown in yellow. Light blue marks non-significant changes in dVSD(t)/dt, here between −3 and 3 10^−7^. NINH in blue and dark blue, NEX in green yellow, beige red.

Movie 5 **Space-time dynamics of statistically significant NEX and NINH (FDR methods < 0.05) in one animal to a scene with a bar moving up from the CFOV**. The color scale values should be multiplied with 10^−6^. Light blue marks non-significant changes in dVSD(t)/dt, here between −4 and 4 10^−7^. Other conventions as in Movie 4.

Movie 6 **Space-time dynamics of statistically significant NEX and NINH (FDR methods < 0.05) in one animal to a scene with a bar moving down from the peripheral field of view**. The color scale values should be multiplied with 10^−6^. Light blue marks non-significant changes in dVSD(t)/dt, here between −7 and 7 10^−7^. Other conventions as in Movie 11.

Movie 7 **Space-time dynamics of statistically significant NEX and NINH (FDR methods < 0.05) in one animal to a scene with a bar moving up from the peripheral field of view**. The color scale values should be multiplied with 10^−6^. Light blue marks non-significant changes in dVSD(t)/dt, here between −10 and 10 10^−7^. Other conventions as in Movie 11.

Movie 8 **State-space displays of trajectories of spatial principal components (PC) of VSD(t) of all visual scenes for animal 2**. The panels are 6 projections of the 4-dimensional state-space explaining more than 94% of the total variance. Color codes: blue, scene with bar moving down from the CFOV, green, scene with bar moving up from CFOV; light blue, scene with bar moving down from the peripheral field of view; purple, scene with bar moving up from the peripheral field of view; yellow, scene with stationary bar. Remaining animals had similar good separation of the scenes, especially by principal components 2 and 3.

Movie 9 **State-space displays of trajectories of spatial principal components (PC) of VSD(t) of all 5 visual scenes for animal 3**.

Movie 10 **State-space displays of trajectories of spatial principal components (PC) of VSD(t) of all 6 visual scenes for animal 4**. The 6th scene was two bars moving toward each other to occlude each other at 413 ms (Harvey and Roland, 2013). This condition is now yellow; the stationary object condition now gray.

Movie 11 **State-space displays of trajectories of spatial principal components (PC) of VSD(t) of all 6 visual scenes for animal 5**.

Movie 12 **State-space displays of trajectories of spatial principal components (PC) of VSD(t) of all 6 visual scenes for animal 6**.

Movie 13 **State-space displays of trajectories of spatial principal components (PC) of VSD(t) of all 6 visual scenes for animal 7**.

Movie 14 **State-space displays of trajectories of spatial principal components (PC) of VSD(t) of all 6 visual scenes for animal 8**.

Movie 15 **Space-time dynamics of phase relations of the VSD(t) of network of six animals (Methods)**. The VSD(t) in each channel in the time period shown was normalized to the maximal value of 1. Notice the advance of the VSD(t) increase in areas 19/21 initially.

## Abbreviations

CFOV: center of field of view
FDR: false discovery rate
FOV: field of view
MUA: multi-unit activity
NEX: net excitation
NINH: net inhibition
VSD(t): averaged voltage sensitive dye signal.
